# An observational study of patho-oncological outcomes of various surgical methods in total mesorectal excision for rectal cancer: a single center analysis

**DOI:** 10.1186/s12893-020-0687-1

**Published:** 2020-02-03

**Authors:** Yi-Ting Chen, Ching-Wen Huang, Cheng-Jen Ma, Hsiang-Lin Tsai, Yung-Sung Yeh, Wei-Chih Su, Chee-Yin Chai, Jaw-Yuan Wang

**Affiliations:** 1 Department of Pathology, Kaohsiung Medical University Hospital, Kaohsiung Medical University, Kaohsiung, Taiwan; 2 Graduate Institute of Medicine, College of Medicine, Kaohsiung Medical University, Kaohsiung, Taiwan; 3 Department of Pathology, Faculty of Medicine, College of Medicine, Kaohsiung Medical University, Kaohsiung, Taiwan; 4 Division of Colorectal Surgery, Department of Surgery, Kaohsiung Medical University Hospital, Kaohsiung Medical University, No. 100 Tzyou 1st Road, Kaohsiung, 807 Taiwan; 5 Department of Surgery, Faculty of Medicine, College of Medicine, Kaohsiung Medical University Hospital, Kaohsiung Medical University, Kaohsiung, Taiwan; 6 Division of General and Digestive Surgery, Department of Surgery, Kaohsiung Medical University Hospital, Kaohsiung Medical University, Kaohsiung, Taiwan; 7 Division of Trauma and Surgical Critical Care, Department of Surgery, Kaohsiung Medical University Hospital, Kaohsiung Medical University, Kaohsiung, Taiwan; 8 Institute of Biomedical Sciences, National Sun Yat-Sen University, Kaohsiung, Taiwan; 9 Graduate Institute of Clinical Medicine, College of Medicine, Kaohsiung Medical University, Kaohsiung, Taiwan; 10 Center for Cancer Research, Kaohsiung Medical University, Kaohsiung, Taiwan

**Keywords:** Robotic, Laparoscopic, Open surgery, Total mesorectal excision, Rectal cancer

## Abstract

**Background:**

Total mesorectal excision (TME) with or without neoadjuvant concurrent chemoradiotherapy (CCRT) is the treatment for rectal cancer (RC). Recently, the use of conventional laparoscopic surgery (LS) or robotic-assisted surgery (RS) has been on a steady increase cases. However, various oncological outcomes from different surgical approaches are still under investigation.

**Methods:**

This is a retrospective observational study comprising 300 consecutive RC patients who underwent various techniques of TME (RS, *n* = 88; LS, *n* = 37; Open surgery, *n* = 175) at a single center of real world data to compare the pathological and oncological outcomes, with a median follow-up of 48 months.

**Results:**

Upon multivariate analysis, histologic grade (*P* = 0.016), and stage (*P* < 0.001) were the independent factors of circumferential resection margin (CRM) involvement. The Kaplan-Meier survival analysis determined RS, early pathologic stage, negative CRM involvement, and pathologic complete response to be significantly associated with better overall survival (OS) and disease-free survival (DFS) (all *P* < 0.05). Multivariable analyses observed the surgical method (*P* = 0.037), histologic grade (*P* = 0.006), and CRM involvement (*P* = 0.043) were the independent factors of DFS, whereas histologic grade (*P* = 0.011) and pathologic stage (*P* = 0.022) were the independent prognostic variables of OS.

**Conclusions:**

This study determined that RS TME is feasible because it has less CRM involvement and better oncological outcomes than the alternatives have. The significant factors influencing CRM and prognosis depended on the histologic grade, tumor depth, and pre-operative CCRT. RS might be an acceptable option owing to the favorable oncological outcomes for patients with RC undergoing TME.

## Background

Colorectal cancer (CRC) is the third most common cancer worldwide [1]; one type of CRC is rectal cancer (RC), which is a life-threating disease. The conventional treatment for RC might involve total mesorectal excision (TME) combined with preoperative neoadjuvant concurrent chemo-radiotherapy (CCRT) and postoperative adjuvant chemotherapy. TME is a skill-dependent procedure and plays a crucial prognostic role. Previous studies have revealed that the status of circumferential resection margin (CRM) influences the local recurrence and overall survival [2, 3]. Adequate lymph nodes retrieval also has a close association with prognosis [4]. Therefore, meticulous TME and adequate lymph node dissection are challenges for the surgeons, especially in cases presenting after preoperative CCRT or with severe adhesion or fibrosis besides an advanced disease stage [5].

With the availability of modern medical facilities and the advancement in surgical techniques, minimally-invasive surgery has garnered the reputation of being the ideal treatment compared with open surgery. Besides the conventional laparoscopic surgery (LS), the robotic-assisted surgery (RS) has gradually become an accepted surgical technique that is considered advantageous. Considering the narrow space of pelvic cavity, RS seems to have better operation plane filed of the presacral fascia and deep pelvic dissection without injury, less conversion rate and postoperative complications [6]. Until now, several large, multicenter randomized control studies had only compared LS with open surgery regarding surgery for RC, with findings showing more CRM involvement rates, worse sexual function, and worse prognosis [7]. However, given the increasing number of RS with reportedly favorable CRM involvement [8], the oncological outcomes of these three different surgical methods are still to be defined.

This study retrospectively analyzed a cohort of 300 consecutive patients with RC who underwent TME to evaluate and compare the oncological outcomes and long-time survival of RS, LS, and open surgery in a real world data.

## Methods

### Patient population and clinical data collection

This study was approved by the Institutional Review Board of our hospital (KMUHIRB-E (II)-20,170,182). Overall, 331 consecutive patients with RC patients who were diagnosed with adenocarcinoma and had undergone surgical intervention at our hospital between 2013 and 2016 were enrolled. The enrollment criteria included no previous or concurrent malignancies, no history of previous abdominal surgery for CRC, no evidence of distant metastasis, presence of complete medical record. However, 31 patients without regular clinical follow-up were excluded (RS, *n* = 7; LS, *n* = 5; Open surgery, *n* = 19).

All the patients had colonoscopy and an abdominopelvic computed tomography scan to evaluate the clinical stage preoperatively. The tumor staging was performed according to the staging guidelines published by the American Joint Committee on Cancer (AJCC) [9]. Patients with clinical T3–4 or nodal involvement received preoperative CCRT, as per a previous study [10]. The preoperative studies, preparation, and operative procedures were according to our literature published previously [8, 11, 12]. Then, all patients underwent total mesorectal excision 8–10 weeks following the completion of long-course CCRT. Surgical method was chosen after shared decision-making with surgeons and patients. The dissection was extended downward and upward to the root of the inferior mesenteric artery with high dissection and low ligation by endo clips with preservation of the left colic artery. The inferior mesenteric vein (IMV) was identified and dissected without ligation. The splenic flexure of the colon was not mobilized routinely, if its mobilization was dependent on the tension of the anastomosis [8]. The clinical data of all patients were retrospectively collected by reviewing the medical records. All three groups received the same postoperative care and surveillance, as per previous study principles [13].

### Pathologic evaluation

All specimens analyzed in this study were available as surgical specimens and had been processed according to the standard pathologic procedures [14]. All formalin-fixed and paraffin-embedded tissue blocks were cut into 3-μm sections and, than deparaffinized and rehydrated. The specimen was recorded by pathologists with the necessary associated information, including specimen length, tumor size, proximal margin, distal margin, harvested lymph node number, and completeness of TME. Adequate number of harvested lymph nodes was defined as equal or more than 12 [15].

The hematoxylin and eosin slides were reviewed to confirm the definitive diagnosis and pathologic characteristics, including disease stage, histologic grade, lymphovascular invasion (LVI), perineural invasion (PNI), and CRM distance. CRM involvement was defined as the distance of 1 mm or less from the cancer cells to the circumferential margin. The pathologic tumor stage was also evaluated according to the AJCC system. Tumor regression score (TRS) was also evaluated if the patients received preoperative CCRT according to grading system of College of American Pathologists (CAP). A four-grade scale is recommended and divided into grade 0 (complete response), grade 1 (moderate response), grade 2 (minimal response), and grade 3 (poor response) [9].

### Statistical analysis

All statistical analyses were performed using the Statistical Package for the Social Sciences, version 24.0 (SPSS Inc., Chicago, IL, USA). The correlation between clinicopathological factors and treatment groups were evaluated using the chi-square test for categorical variables and Student’s *t* test for continuous variables. Univariate and multivariable logistic regression models were used to evaluate the independent predictors of CRM involvement. Disease free survival (DFS) and overall survival (OS) were examined using the Kaplan–Meier method, and the log-rank test was used to compare time-to-event distributions. OS was defined as the duration between date of primary treatment and date of death from any cause or to the last follow-up date. DFS was defined as the duration between date of primary treatment date to the date of recurrence or metastasis or to the last follow-up date. A Cox proportional hazard model was used for multivariable analyses to identify the independent prognostic factors for OS and DFS. All tests were two-tailed, and a *P* value of less than 0.05 was considered statistically significant.

## Results

### Clinicopathological factors and postoperative outcomes of different surgical methods

**Fig. 1:**
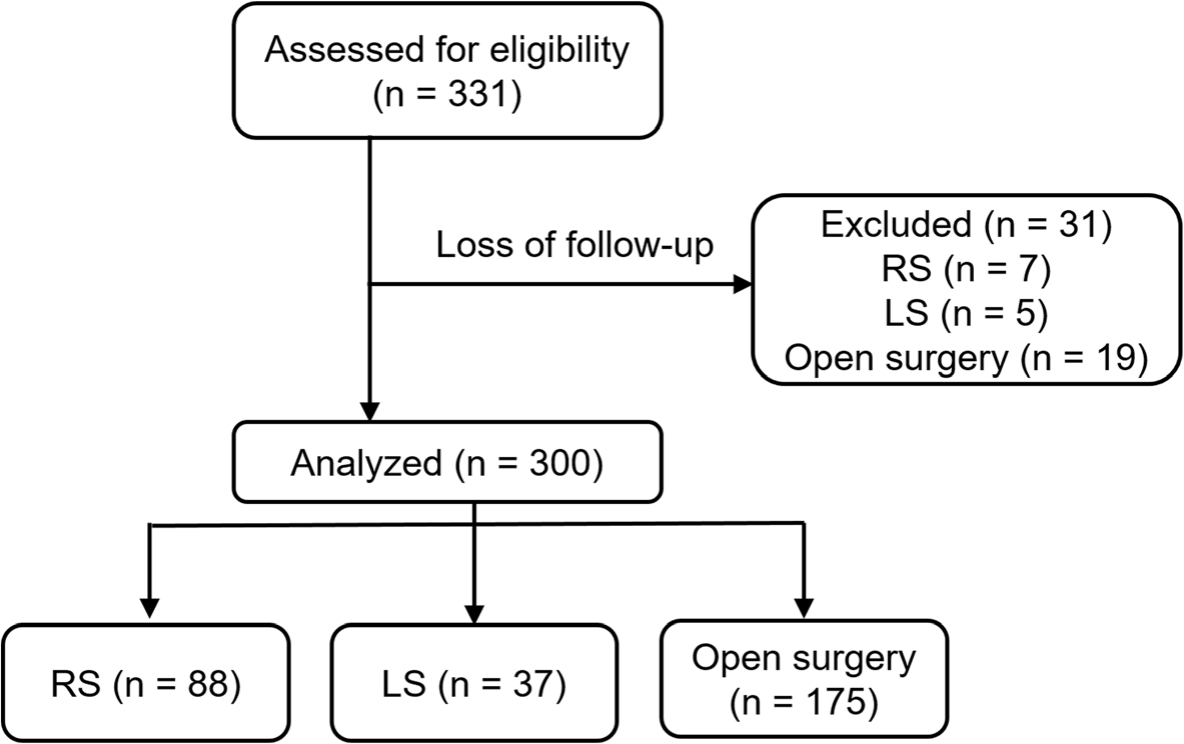
Flow chart of the enrollment of study (RS: robotic surgery; LS: laparoscopic surgery)

Overall, 300 consecutive patients with RC were enrolled in this study, which included 88 patients who underwent robotic surgery, 37 who underwent laparoscopic surgery, and the remaining 175 patients who underwent open surgery (Fig. 1). Patients’ characteristics are shown in Table 1. No intergroup differences related to sex, age, and tumor size were observed (all *P* > 0.05). All specimens were sampled according to standard procedure. The median follow-up period was 48 ± 16.6, 47.5 ± 22.7, and 48 ± 17.8 months for the RS, LS, and open surgery groups, respectively.

**Table 1:** Baseline characteristics and pathologic outcomes of rectal cancer patients in RS, LS and open surgery groups

Variable	n	RS (%)	LS (%)	Open surgery (%)	*P* value
All	300	*N* = 88 (29.33%)	37 (12.33%)	175 (58.33%)	
Age (years, median ± SD)		62 ± 12.1	60 ± 11.1	64 ± 10.4	0.058
Gender					0.253
male	192 (64%)	54 (61.4%)	20 (54.1%)	118 (67.4%)	
female	108(36%)	34 (38.6%)	17(45.9%)	57 (32.6%)	
Tumor size					0.201
> 5 cm	55 (18.3%)	11 (12.5%)	9 (24.3%)	35 (20%)	
≦5 cm	245 (81.7%)	77 (87.5%)	28 (76.7%)	140 (80%)	
Distance to resection margin (cm, median ± SD)					
proximal		6.5 ± 3.3	8.8 ± 4.1	5.5 ± 4.1	0.038*
distal		2.3 ± 1.5	2.5 ± 2.4	2.2 ± 1.9	0.679
Distal resection margin					0.794
> 2 cm	182 (60.7%)	56 (63.6%)	22 (59.5%)	104 (59.4%)	
≦2 cm	118 (39.3%)	32 (36.4%)	15 (40.5%)	71 (40.6%)	
Number of retrieval LN (median ± SD)		9.0 ± 5.3	13.0 ± 6.6	14.0 ± 6.6	< 0.001*
Retrieval lymph nodes					0.417
Adequate	274 (91.3%)	78 (88.6%)	33 (89.3%)	163 (93.1%)	
inadequate	26 (8.7%)	10 (11.4%)	4 (10.7%)	12 (6.9%)	
Pre-operative CCRT					< 0.001*
yes	156 (52%)	72 (81.8%)	15 (40.5%)	69 (39.4%)	
no	144 (48%)	16 (18.2%)	22 (59.5%)	106 (60.6%)	
Histologic grade					0.158
WD	35 (11.7%)	14 (15.9%)	2 (5.4%)	19 (10.9%)	
MD	251 (83.7%)	67 (76.1%)	34 (91.9%)	150 (85.7%)	
PD	14 (4.7%)	7 (8%)	1 (2.7%)	6 (3.4%)	
LVI					0.006*
yes	109 (36.3%)	21 (23.9%)	19 (51.4%)	69 (39.4%)	
no	191 (63.7%)	67 (76.1%)	18 (48.6%)	106 (60.6%)	
PNI					0.058
yes	70 (23.3%)	16 (18.2%)	14 (37.8%)	40 (22.9%)	
no	230 (76.7%)	72 (81.8%)	23 (62.2%)	135 (77.1%)	
CRM (mm, median ± SD)		8.0 ± 6.4	5.0 ± 7.8	5.0 ± 7.2	0.118
CRM involvement					0.037*
yes	30 (10%)	3 (3.4%)	6 (16.2%)	21 (12%)	
no	270 (90%)	85 (96.6%)	31 (83.8%)	154 (88%)	
pStage (AJCC 7th edition)					0.014*
pCR	50 (16.7%)	24 (27.3%)	5 (13.5%)?	21 (12%)	
I	82 (27.3%)	27 (30.7%)	9 (24.3%)	46 (26.3%)	
II	73 (24.3%)	17 (19.3%)	7 (18.9%)	49 (28%)	
III	95 (31.7%)	20 (22.7%)	16 (43.2%)	59 (33.7%)	
Tumor depth					0.005*
(y)pT0	53 (17.7%)	26 (29.5%)	5 (13.5%)	22 (12.6%)	
(y)pT1	31 (10.3%)	13 (14.8%)	2 (5.4%)	16 (9.1%)	
(y)pT2	71 (23.7%)	19 (21.6%)	11 (29.7%)	41 (23.4%)	
(y)pT3	145 (48.3%)	30 (34.1%)	19 (51.4%)	96 (54.9%)	
Lymph node metastasis					0.041*
(y)pN0	205 (68.3%)	68 (77.3%)	21 (56.8%)	116 (66.3%)	
(y)pN1	66 (22%)	18 (20.5%)	10 (27%)	38 (21.7%)	
(y)pN2	29 (9.7%)	2 (2.2%)	6 (16.2%)	21 (12%)	
Tumor regression score after CCRT					0.784
0	50 (32.1%)	24 (33.3%)	5 (33.3%)	21 (30.4%)	
1	61 (39.1%)	32 (44.4%)	5 (33.3%)	24 (34.8%)	
2	34 (21.8%)	12 (16.7%)	4 (26.7%)	18 (26.1%)	
3	11 (7.1%)	4 (5.6%)	1 (6.7%)	6 (8.7%)	
Pathologic complete response after CCRT					0.928
yes	50 (32.1%)	24 (33.3%)	5 (33.3%)	21 (30.4%)	
no	106 (67.9%)	48 (66.7%)	10 (66.7%)	48 (69.6%)	
Post-operative distant metastasis					0.151
yes	68 (22.7%)	19 (21.6%)	13 (35.1%)	36 (20.6%)	
no	232 (77.3%)	69 (78.4%)	24 (64.9%)	139 (79.4%)	
Post-operative local recurrence					< 0.001*
yes	22 (7.3%)	2 (2.3%)	8 (21.6%)	12 (6.9%)	
no	278 (92.7%)	86 (97.7%)	29 (78.4%)	163 (93.1%)	

*RS* robotic surgery; *LS* laparoscopic surgery; *LN* lymph node; *WD* well-differentiated; *MD* moderately-differentiated; *PD* poorly-differentiated; *LVI* lymphovascular invasion; *PNI* perineural invasion; *LN* lymph node; *CRM* circumferential margin; *AJCC* American Joint Commission on Cancer; *pCR* pathologic complete response; *CCRT* concurrent chemoradiotherapy; *P **< 0.05**

Overall, the distance to proximal or distal resection margins, TME completeness status, and retrieved lymph node number were evaluated by pathologists. No significant differences were observed regarding distance to distal resection margin, TME specimen status, and adequacy of lymph node removal (Table 1, all *P* < 0.05). Longer distance to proximal resection margin was identified in LS group (*P* = 0.004). Notably, the distance to proximal resection margin was longer in LS (8.8 ± 4.1 cm) compared with RS (6.5 ± 3.3 cm) and open surgery (5.5 ± 4.1 cm) groups (*P* = 0.038). Lesser number of retrieved lymph nodes (9.0 ± 5.3) was seen in RS group (*P* = 0.000) compared with LS (13.0 ± 6.6) and open surgery (14.0 ± 6.6) groups. However, no differences were observed in the adequacy of lymph node retrieval between these three methods (*P* = 0.431).

Histologic grade, LVI, PNI, CRM status, and pathologic stage were assessed microscopically. Tumor regression grade was also investigated in 156 patients who received preoperative CCRT. No differences were observed regarding histologic grade, PNI, CRM distance, TRS, CR, and postoperative metastasis between these groups (all *P* > 0.05). LVI and CRM involvement was more in the LS group (*P* = 0.006 and *P* = 0.037, respectively). Advanced pathologic stage (*P* = 0.018), deeper tumor invasion (*P* = 0.005), more lymph node metastasis (*P* = 0.041), and postoperative local recurrence (*P* = 0.001) were also noted more frequently in the LS group. Postoperative local recurrence was significantly lower in the RS group compared with the LS and open surgery groups (*P* < 0.001), albeit with no differences regarding the postoperative distant metastasis (Table 1).

### CRM involvement associated with other factors

Concerning the significance of CRM, Table 2 shows the CRM status in relation to other clinicopathological parameters. Of the 300 patients with RC, 30 (10%) presented with CRM involvement by cancer cells. Pearson’s correlation coefficients were calculated to analyze the relationships between CRM status and clinicopathologic factors. No differences were observed related to age, sex, and CR between CRM involvement and non-involvement groups (all *P* > 0.05). Regarding the surgery methods, of 37 patients in the LS group, 6 (16.2%) showed positive CRM involvement compared with 3 (0.3%) of 88 cases in the RS group and 21 (12%) of 175 cases in the open surgery group (*P* = 0.037). Larger tumor size (*P* < 0.001), poorly-differentiated tumor cells (*P* = 0.001), presence of LVI (*P* < 0.001), and PNI (*P* = 0.023) were associated with CRM involvement. Additionally, advanced pathologic stage and tumor stage were also significantly associated with CRM involvement (both *P* < 0.001). More CRM involvement was noted in patients with RC who had no preoperative CCRT and higher TRS (*P* = 0.002).

**Table 2:** Baseline characteristics and pathologic outcomes of rectal cancer patients by CRM involvement status

Variable	n	CRM involvement		
		Yes	No	
All	300	30 (10%)	270 (90%)	*P* value
Age		60.0 ± 12.4	63.0 ± 10.9	0.185
Gender				0.262
female	108 (36%)	8 (7.4%)	100 (92.6%)	
male	192 (64%)	22 (11.5%)	170 (88.5%)	
Surgery method				0.037*
RS	88 (29.3%)	3 (3.4%)	85 (96.6%)	
LS	37 (12.3%)	6 (16.2%)	31 (83.8%)	
Open surgery	175 (58.3%)	21 (12%)	154 (88%)	
Tumor size				< 0.001*
> 5 cm	55 (18.3%)	14 (25.5%)	41 (74.5%)	
≦5 cm	245 (81.7%)	16 (6.5%)	229 (93.5%)	
Histologic grade				0.001*
WD	35 (11.7%)	0 (0%)	35 (100%)	
MD	251 (83.7%)	25 (10.0%)	226 (90.0%)	
PD	14 (4.7%)	5 (35.7%)	9 (64.3%)	
LVI				< 0.001*
yes	109 (36.3%)	24 (22.0%)	85 (78.0%)	
no	191 (63.7%)	6 (3.1%)	185 (96.9%)	
PNI				0.023*
yes	70 (23.3%)	12 (17.1%)	58 (82.9%)	
no	230 (76.7%)	18 (7.8%)	212 (92.2%)	
Stage				< 0.001*
pCR	50 (16.7%)	0 (0%)	50(100%)	
I	82 (27.3%)	1 (1.2%)	81 (98.8%)	
II	73 (24.3%)	7 (9.6%)	66 (90.4%)	
III	95 (31.7%)	22 (23.2%)	73 (76.8%)	
Tumor depth				< 0.001*
(y)pT0	53 (17.7%)	0 (0%)	53 (100%)	
(y)pT1	31 (10.3%)	0 (0%)	31 (100%)	
(y)pT2	71 (23.7%)	1 (1.4%)	70 (98.6%)	
(y)pT3	145 (48.3%)	29 (20%)	116 (80%)	
Pre-OP CCRT				< 0.001*
yes	156 (52%)	4 (2.6%)	152 (97.4%)	
no	144 (48%)	26 (18.1%)	118 (81.9%)	
TRS after CCRT	156	4 (2.6%)	152 (97.4%)	0.002*
0	50 (32.05%)	0 (0%)	50 (100%)	
1	61 (29.10%)	0 (0%)	61 (100%)	
2	34 (21.9%)	2 (5.9%)	32 (94.1%)	
3	11 (7.1%)	2 (18.2%)	9 (81.8%)	
CR after CCRT	156	4 (2.6%)	152 (97.4%)	0.164
yes	50 (32.1%)	0 (0%)	50 (100%)	
no	106 (67.9%)	4 (3.8%)	102 (96.2%)	

*CRM* circumferential margin; *SD* standard deviation; *OR* odds ratio; *CI* confidence interval; *RS* robotic surgery; *LS* laparoscopic surgery; *CCRT* concurrent chemoradiotherapy; *WD* well-differentiated; *MD* moderately-differentiated; *PD* poorly-differentiated; *LVI* lymphovascular invasion; *PNI* perineural invasion; *AJCC* American Joint Commission on Cancer; *pCR* pathologic complete response; *OP* operative, *TRS* tumor regression score; *P < 0.05

The logistic regression modelling was used to assess odds ratio of CRM status using clinicopathologic factors (Table 3). Upon univariate analysis, the CRM involvement was associated with the surgery method, histologic grade (*P* = 0.003), and stage (*P <* 0.001). The multivariate result showed that histologic grade (*P* = 0.016) and stage (*P <* 0.001) were the independent factors of CRM involvement.

**Table 3:** Univariate and multivariable analysis to predict CRM involvement for rectal cancer patients

	Univariate		Multivariate	
Parameters	OR (95% CI)	*P* value	OR (95% CI)	*P* value
Surgery method		0.057		0.069
RS vs. LS	1.292–23.276	0.021*	1.17–18.87	0.029*
RS vs. Open surgery	1.120–13.329	0.032*	0.435–3.62	0.674
Histologic grade (WD + MD vs. PD)	1.804–18.644	0.003*	1.38–22.22	0.016*
Stage (0-II vs. III)	3.164–17.408	< 0.001*	2.44–14.08	< 0.001*

*CRM* circumferential margin; *SD* standard deviation; *OR* odds ratio; *CI* confidence interval; *RS* robotic surgery; *LS* laparoscopic surgery; *WD* well-differentiated; *MD* moderately-differentiated; *PD* poorly-differentiated, *P < 0.05

### Prognostic values in RC patients

**Fig. 2:**
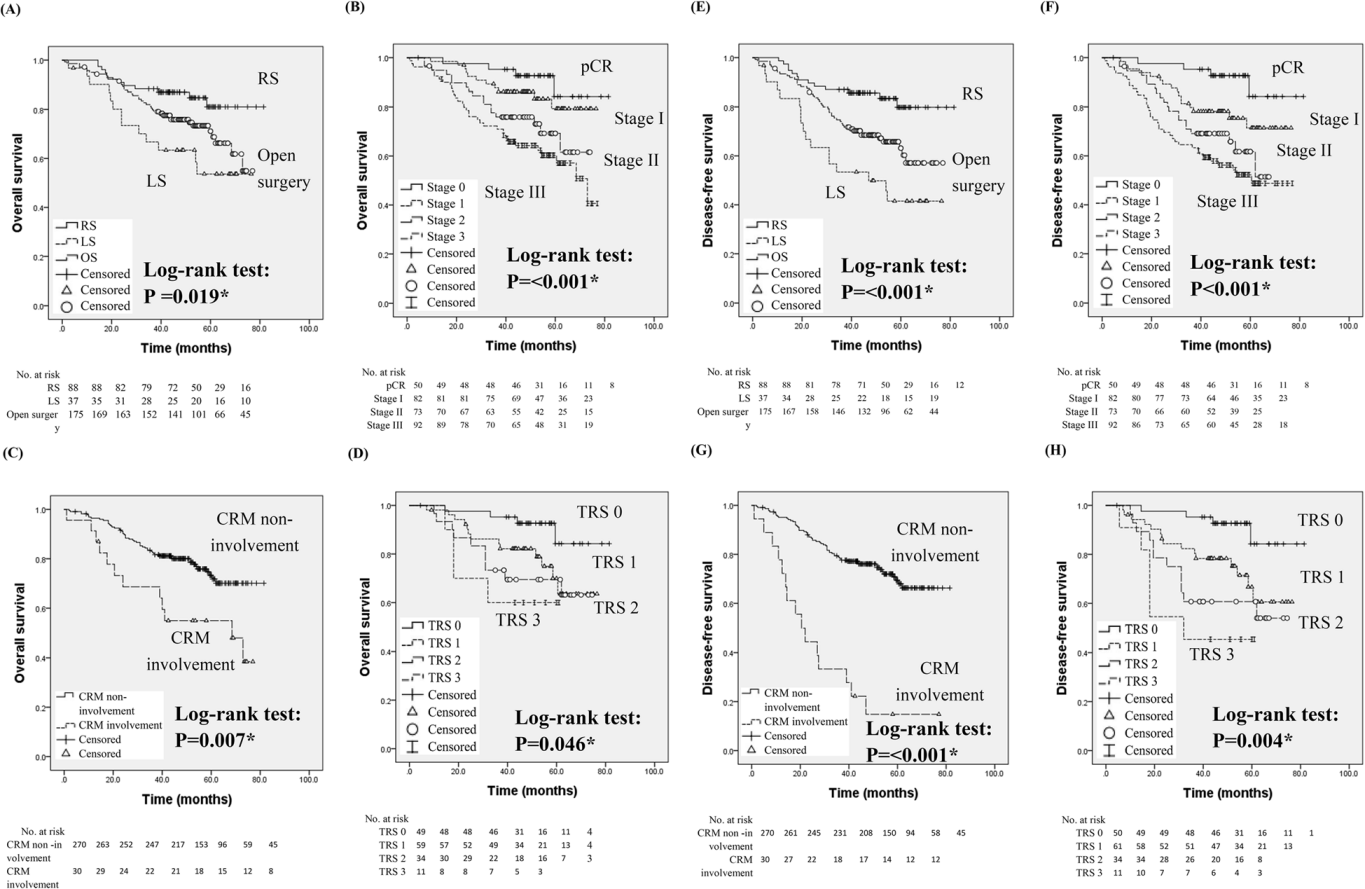
Kaplan-Meier method analyzed overall survival of (**a**) surgical methods (RS: robotic surgery; LS: laparoscopic surgery; open surgery), (**b**) tumor stage (pCR: pathologic complete response), (**c**) CRM (circumferential resection margin) status, (**d**) tumor regression score (TRS). Kaplan-Meier method analyzed disease-free survival of: (**e**) surgical methods, (**f**) tumor stage, (**g**) CRM status, and (**h**) TRS

Figure 2 revealed that LS was significantly associated with poor OS (*P* = 0.019); conversely, early stage (*P* < 0.001), non-CRM involvement (*P* = 0.007), and lower TRS (*P* = 0.046) after CCRT were associated with better OS. 12, 13 and 40 patients receiving RS, LS and open surgery expired in the subsequent follow-up period. The overall survival rate is 86.4, 64.9 and 77.1%, respectively. The results showed that LS (*P* < 0.001), advanced pathologic stage (*P* < 0.001), positive CRM involvement (*P* < 0.001), and TRS (*P* = 0.004) was associated with worse DFS.

Upon multivariable analyses, the results showed that surgical method (*P* = 0.037), histologic grade (*P* = 0.006), and CRM involvement (*P* = 0.043) were the independent prognostic factors of DFS in patients with RC, whereas histologic grade (*P* = 0.011) and pathologic stage (*P* = 0.022) were independent prognostic factors of OS (Table 4).

**Table 4:** Univariate and multivariate analysis of prognostic indicators to predict disease-free survival and overall survival for rectal cancer patients

	Disease-free survival				Overall survival			
	Univariate analysis		Multivariate analysis		Univariate		Multivariate	
Covariate	HR (95% CI)	*P* value	HR (95% CI)	*P* value	HR (95% CI)	*P* value	HR (95% CI)	*P* value
Gender (female vs. male)	0.536–1.358	0.504	0.332–1.567	0.409	0.546–1.496	0.694	0.294–1.632	0.401
Age (≦65 y/o vs. > 65 y/o)	0.532–1.313	0.436	0.681–3.732	0.283	0.450–1.194	0.212	0.296–1.729	0.457
Surgical method		0.001*		0.037*		0.024*		0.127
RS vs. LS	1.176–4.016	0.013*	1.163–6.024	0.020*	0.846–2.967	0.015*	1.033–6.132	0.042*
RS vs. Open surgery	1.071–3.261	0.028*	0.457–3.268	0.690	0.279–1.015	0.055	0.492–6.384	0.382
Tumor size (≦5 vs. > 5 cm)	1.111–3.303	0.018*	0.614–12.201	0.187	0.380–1.174	0.161	0.090–2.001	0.279
CRM involvement (no vs. yes)	2.505–7.788	< 0.001*	1.053–23.806	0.043*	1.236–4.427	0.009*	0.563–48.181	0.146
Tumor grade (WD/MD vs. PD)	1.345–6.403	0.007*	1.717–27.106	0.006*	1.143–6.217	0.023*	1.554–28.879	0.011*
LVI (no vs. yes)	1.357–3.318	0.001*	0.015–11.118	0.596	1.441–3.834	0.001*	0.012–1.890	0.144
PNI (no vs. yes)	1.699–4.288	< 0.001*	0.998–6.835	0.051	1.734–4.740	< 0.001*	0.625–5.266	0.273
pT stage (T1–2 vs. T3)	1.5744.502	< 0.001*	0.269–1.665	0.388	1.505–4.259	< 0.001*	0.290–2.202	0.665
Pathologic stage (stage 0-II vs. III)	1.429–3.496	< 0.001*	0.230–155.766	0.282	1.480–3.919	< 0.001*	1.497–178.914	0.022*
TRS (0–1 vs. 2–3)	1.331–4.864	0.005*	0.626–2.831	0.457	1.014–4.199	0.046*	0.400–2.157	0.864
pCR (yes vs. no)	1.563–12.478	0.005*	0.117–1.304	0.127	0.101–0.828	0.021*	0.135–1.585	0.220

*CI* confidence interval; *RS* robotic surgery; *LS* laparoscopic surgery; *CRM* circumferential margin; *WD* well-differentiated; *MD* moderately-differentiated; *PD* poorly-differentiated; *LVI* lymphovascular invasion; *PNI* perineural invasion; *LN* lymph node; *OP* operative; *TRS* tumor regression score; *pCR* pathological complete response; *P < 0.05

## Discussion

RC is a crucial cause of cancer-related deaths worldwide. Preoperative or postoperative chemoradiotherapy is the gold standard treatment for RC. Moreover, TME is a crucial procedure in the surgical treatment of RC. However, TME is a skill-dependent procedure and plays a critical role in the prognosis. Previous studies have shown that complete TME with adequate circumferential margin (CRM) significantly affects the OS and local recurrence [2, 3].

The first laparoscopic colectomy was performed in 1991 [16], which was then performed increasingly by surgeons as an alternative intervention for achieving better short-term outcome compared with open surgery. Nevertheless, laparoscopic rectal TME is challenging owing to the limited anatomic pelvic surgical plane, rigidity of scope, hand tremor of camera-holding assistant, and resolution of two-dimensional visualization. This shortcoming paved way for the first robotic colon surgery in 2002, and this new minimally-invasive system tried to overcome the disadvantages of the conventional LS and improve the clinical outcomes. Additionally, the learning curve for RS is reported to be lesser than conventional LS [16, 17]. In current modern medicine, robotic surgery is considered as a revolutionary procedure and one of the best treatment options for patients with RC. Previous reviews have shown that robotic-assisted intervention has significant benefits in rectal surgery. Moreover, it can also preserve urinary and sexual functions [18]. Notably, robotic intervention has been widely used in various cancer surgeries. Therefore, it is imperative to address the oncological outcomes of the three different surgical TME methods in patients with RC, namely RS, LS, and open surgery.

Our study included 300 consecutive patients with RC, and no intergroup differences related to age and sex were observed. The LS group had longer proximal resection margin; however, no differences were observed regarding distal margin, TME status, and adequacy of lymph node retrieval, which was concordant with previous literatures [19–21]. Regarding the number of harvested lymph nodes, a lesser amount was noted in the RS group, of which was the same as that observed by Lee et al. [22]. The possible reason for this finding was a higher proportion of patients receiving preoperative CCRT in this group. A previous study had revealed that neoadjuvant chemotherapy was a significant factor for inadequate harvesting of lymph nodes in colon cancer owing to lymphocyte destruction and post-radiation fibrosis [23]. On the other hand, fewer than 12 lymph nodes retrieved in patients with RC who underwent neoadjuvant radiotherapy was considered to be an excellent indicator of tumor response, better local lesion control, and a positive prognostic factor. Upon comparing the adequacy of lymph node retrieval, no differences were observed between the three methods. Microscopically, no differences were noted regarding the histologic grade, PNI, and CRM distance between the three groups. In addition, LVI and advanced stage were observed in the LS group, probably because of fewer patients receiving preoperative CCRT in the LS group because neoadjuvant therapy can decrease the size of tumor cells, besides downstaging or even causing complete remission after the treatment [24, 25]. However, no differences were noted regarding TRS and CR among the three groups. More postoperative recurrence was also noted in the LS group, which may be related to more CRM involvement. Consistent with previous literatures, the evidences also showed the non-inferiority of LS compared with open surgery for clear CRM and complete TME was not established or supported [26, 27].

Concerning the role of CRM in patients with RC, 10% of our patients had CRM involvement by tumors. More CRM involvement was identified` in the LS group, larger tumor size, poorly-differentiated histologic grade, presence of LVI/PNI, advanced pathologic stage, and deeper tumor invasion. Patients with preoperative CCRT and lower TRS after CCRT were associated with lesser CRM involvement. However, after logistic regression modelling, the results showed that histologic grade, tumor depth stage, and preoperative CCRT were the independent factors of CRM involvement. Likewise, Nikberg et al. reported that more CRM involvement was noted in patients with advanced stage [28]. Accordingly, advanced stage was considered the most crucial factor of CRM involvement in patients with RC.

Upon Kaplan-Meier survival analysis, no significance was noted related to age, sex, tumor size, resection margin (proximal and distal), CCRT status, number of lymph nodes retrieved, and histologic grade (data not shown). However, surgical methods, advanced pathologic stage, CRM involvement, and TRS after CCRT were all identified to be significantly associated with OS. The results were concordant with several previous studies [21]. Kim et al. reported that RS had a significant prognostic role for OS and cancer-specific survival, thereby suggesting its potential oncological benefits; however, the final data showed that histologic grade and pathologic stage were the independent prognostic markers in patients with RC [29]. Furthermore, we evaluated the parameters for predicting recurrence by DFS, and it showed surgical methods, CRM involvement, and histologic grade were independent factors for recurrence in these patients. Our RS group had significantly lower postoperative local recurrence and better DFS compared with other groups, which was consistent with a previous 54-month follow-up study conducted by Yamaguchi et al. [30]. The probable reason for this may be the less CRM involvement in RS patients. Ghezzi et al. also demonstrated that their RS group had a relatively low cumulative local recurrence rate [19]; conversely, several studies showed no differences among these groups, but all of them had enrolled few patients or had shorter follow-up periods [31–34].

Recent literatures have revealed that less estimated blood loss, faster recovery time, and shorter length of postoperative stay were observed in RS compared with open surgery [19, 34, 35]. Considering the limited place in the pelvic area, RS can provide tridimensional view, tremor filtering, better image resolution, and wider operative plane owing to the improved technique. RS results in bloodless surgery, less postoperative pain, fewer conversion rates, nerve-sparing, lower overall complications, and fewer rates of CRM involvement compared with than LS [19, 36–39]. Furthermore, RS contributes to faster return of bowel movements and oral intake, both of which decrease the postoperative hospital stay [35, 40]. Thus, based on our investigation, the oncological outcome of RS was comparatively better than that of LS or open surgery, thereby providing better quality of treatment. Therefore, robotic-assisted surgery could be a suitable treatment option in patients with RC.

Nonetheless, this cohort study had some limitations. First, it was not a randomized-control trial and the clinicopathologic analysis was performed retrospectively. Patients without regular follow were excluded, which may cause selection bias. Second, although no differences were noted related to age and sex in these three methods, fewer patients were enrolled in LS group, and a higher ratio of CCRT and pathologic complete response was noted in the RS group, which may be the reason that more positive CRM was identified in LS group. Therefore, a hidden bias may exist that might influence other factors statistically. For adjusting the selection biases, we used logistic regression modelling of multivariate analysis to analyze the CRM status and prognosis factors. Tumor stage and histologic grade were confirmed to play the most significant role in CRM involvement. Third, the clinical data of operative or post-operative complications were not analyzed together in this study. The complication rate of these three surgical methods in our hospital is around 21%, including bleeding, intra-abdominal abscess or infection, ileus, anastomosis leakage, urethral injury and pulmonary complication according to our previous research [41]. But most of complications can be managed by conservative treatment; therefore, it would not clearly affect the timing of adjuvant chemotherapy.

## Conclusions

Robotic-assisted TME probably provides a favorable local disease control rate and DFS without compromising on the oncological outcomes compared with LS or open surgery method. Nevertheless, robotic surgery might be an acceptable option that could be a beneficial surgical intervention in patients with RC.

## Data Availability

No applicable.

## Abbreviations

AJCC: American Joint Committee on Cancer
CCRT: Concurrent chemoradiotherapy
CRC: Colorectal cancer
CRM: Circumferential resection margin
DFS: Disease-free survival
LS: Laparoscopic surgery
OS: Overall survival
RC: Rectal cancer
RS: Robotic-assisted surgery
TME: Total mesorectal excision
TRS: Tumor regression score
